# Survey on helminths of bats in the Yucatan Peninsula: infection levels, molecular information and host–parasite networks

**DOI:** 10.1017/S0031182022001627

**Published:** 2023-02

**Authors:** Wilson I. Moguel-Chin, David I. Hernández-Mena, Marco Torres-Castro, Roberto C. Barrientos-Medina, Silvia F. Hernández-Betancourt, M. Cristina MacSwiney G., Luis García-Prieto, Víctor M. Vidal-Martínez, Celia Isela Selem-Salas, Jesús Alonso Panti-May

**Affiliations:** 1 Facultad de Medicina Veterinaria y Zootecnia, Campus de Ciencias Biológicas y Agropecuarias, Universidad Autónoma de Yucatán, km 15.5 carretera Mérida-Xmatkuil, Mérida 97135, Yucatán, Mexico; 2 Centro de Investigación y de Estudios Avanzados, Instituto Politécnico Nacional, Unidad Mérida, Carretera Mérida-Progreso, Loma Bonita, Mérida 97205, Yucatán, Mexico; 3 Centro de Investigaciones Regionales ‘Dr. Hideyo Noguchi’, Universidad Autónoma de Yucatan, Av. Itzáes, Centro, Mérida 97000, Yucatán, Mexico; 4 Centro de Investigaciones Tropicales, Universidad Veracruzana, José María Morelos y pavón 44, Centro, Xalapa 91000, Veracruz, Mexico; 5 Instituto de Biología, Universidad Nacional Autónoma de México, Ciudad Universitaria 04510, Ciudad de México, Mexico

**Keywords:** Cestoda, chiropters, host–parasite interactions, molecular analysis, morphology, Nematoda, Trematoda

## Abstract

Helminth species of Neotropical bats are poorly known. In Mexico, few studies have been conducted on helminths of bats, especially in regions such as the Yucatan Peninsula where Chiroptera is the mammalian order with the greatest number of species. In this study, we characterized morphologically and molecularly the helminth species of bats and explored their infection levels and parasite–host interactions in the Yucatan Peninsula, Mexico. One hundred and sixty-three bats (representing 21 species) were captured between 2017 and 2022 in 15 sites throughout the Yucatan Peninsula. Conventional morphological techniques and molecular tools were used with the 28S gene to identify the collected helminths. Host–parasite network analyses were carried out to explore interactions by focusing on the level of host species. Helminths were found in 44 (26.9%) bats of 12 species. Twenty helminth taxa were recorded (7 trematodes, 3 cestodes and 10 nematodes), including 4 new host records for the Americas. Prevalence and mean intensity of infection values ranged from 7.1 to 100% and from 1 to 56, respectively. Molecular analyses confirmed the identity of some helminths at species and genus levels; however, some sequences did not correspond to any of the species available on GenBank. The parasite–host network suggests that most of the helminths recorded in bats were host-specific. The highest helminth richness was found in insectivorous bats. This study increases our knowledge of helminths parasitizing Neotropical bats, adding new records and nucleotide sequences.

## Graphical abstract

**Figure:**
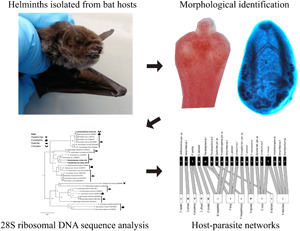

## Introduction

Chiroptera is one of the most speciose, diverse and widespread mammalian orders worldwide, with nearly 1450 species (Simmons and Cirranelo, 2022). Particularly, in the Neotropical zone 450 species have been recorded (Díaz *et al*., 2021). Despite this high diversity, the study of helminth communities of this host group is relatively rare compared to those of other wild mammals (Lord *et al*., 2012). The most recent gathering of information on the helminth–bat association carried out in the Neotropical zone confirms this assertion: the number of helminth taxa registered in 27 species of bats analysed in Mexico and Central America is 68 (Jiménez *et al*., 2017), while in South American bats, Santos and Gibson (2015) reported 114 nominal species of helminths from 92 named bat taxa.

In Mexico, studies on bat helminths began in the late 1930s (Chitwood, 1938; Stunkard, 1938), totalizing 53 helminth species recorded to date (Jiménez *et al*., 2017; Salinas-Ramos *et al*., 2017; Luviano-Hernández *et al*., 2018; Panti-May *et al*., 2021). On the other hand, the number of bats surveyed for helminths in the country represents roughly 14% of the richness of chiropterans present (Jiménez *et al*., 2017). Particularly, in the Yucatan Peninsula (formed by the Mexican states of Campeche, Yucatan and Quintana Roo) 64 species of bats are distributed, which are included in 7 families (Sosa-Escalante *et al*., 2013, 2014). However, only 11 helminth taxa have been reported in Yucatan and 3 in Campeche from 6 bat species (Jiménez *et al*., 2017; Panti-May *et al*., 2021). The remaining 58 bat species have not been studied from a helminthological perspective.

The majority of data on helminths of bats in Mexico come from checklists of species, new species descriptions and new hosts or locality records based on morphological identifications, whereas molecular characterization is limited to the work of Panti-May *et al*. (2021). Worldwide completeness is uncommon in host–helminth inventories (Poulin and Presswell, 2016). To accelerate the rate of description of helminth biodiversity, an integrative taxonomy approach combining traditional morphological description and modern molecular genetics is necessary to characterize helminth species (Poulin and Presswell, 2016; Poulin *et al*., 2019). The increased availability of gene sequences of helminths in the last 2 decades has also proven essential for progress in resolving several crucial elements towards a clear understanding of the process of host–parasite coevolution, such as parasite phylogeny, cryptic parasite diversity and gene flow among parasite populations (Poulin *et al*., 2019).

In addition to the integrative taxonomy, carrying out ecological studies in the host populations, such as network analysis, will allow determining some factors associated with the transmission of parasites between hosts (Luis *et al*., 2015; Runghen *et al*., 2021); this, in turn, will provide information on the potential hosts for some helminth groups. Certain ecological attributes of hosts (locomotion, diet, activity period, etc.) and parasites (type of life cycle) would increase their chance of acquiring parasite infections; however, the number of possible host–parasite interactions may be limited by phylogeny to a subset of species with a shared co-evolutionary history (Poulin, 2010; Pilosof *et al*., 2015). Therefore, to understand the ecology and evolution of both parasites and their hosts, it is important to realize ecological studies at different levels (individual, populations and communities), in which the patterns of interaction between hosts and parasites are evaluated (Bellay *et al*., 2018; Paladsing *et al*., 2020). In this context, the objective of the present study was divided into several folds: (1) to establish the helminth fauna of bats using an integrative taxonomy approach (morphological characters and phylogenetic analysis); (2) to characterize their infection levels and (3) to explore the helminth–bat interactions through network analysis in the Yucatan Peninsula, Mexico.

## Materials and methods

### Collection and examination of bats

**Fig. 1.**
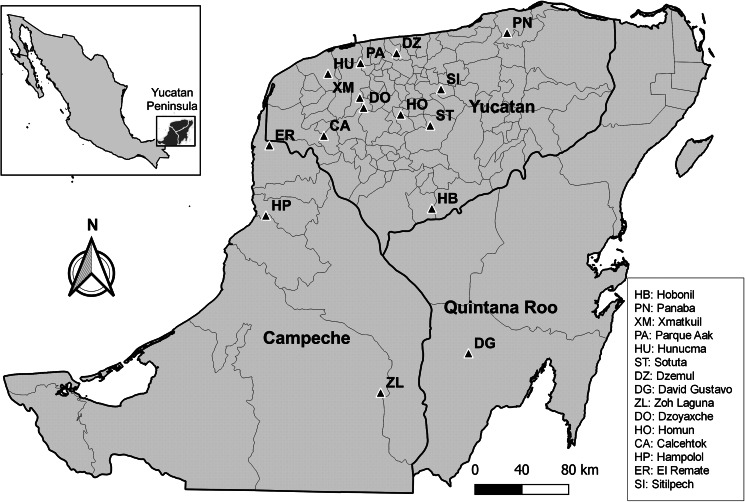
Location of the sites (black triangles) where bats were captured in the Yucatan Peninsula, Mexico.

Bats were collected from 2017 to 2022 in 15 sites throughout the Yucatan Peninsula (Fig. 1), including cattle ranches, natural parks and ecotourism hotels under permits from the Mexican Ministry of Environment (SGPA/DGVS/03705/17, SGPA/DGVS/001643/18, SGPA/DGVS/05995/19, SGPA/DGVS/00786/21 and 31/K5-0032/02/22). In each collection site, 1 or 2 mist nests (12 m wide × 2.5 m high) were placed close to vegetation, natural ponds, animal pens or cave entrances, for 1–2 nights (Table 1). The captured bats were removed from the nets, placed in cloth bags and identified using the field guide of Medellín *et al*. (2008). Animals were anaesthetized with isoflurane and euthanized by overdose of sodium pentobarbital. The heart, lungs, stomach, liver, intestines and mesenteries of each specimen were collected and stored in 96% ethanol.

**Table 1.** Sample sites of bats examined in this study

Site	Municipality, state	Coordinates	Mist net site	Date
Hobonil (HB)	Tzucacab, Yucatan	20°00′59.27″N, 89°01′12.65″W	Tropical forest	March 2017
Hampolol (HP)	Hampolol, Campeche	19°56′30.75″N, 90°22′30.85″W	Near a natural pond	May 2017
Panaba (PN)	Panaba, Yucatan	21°22′49.93″N, 88°25′03.87″W	Near an animal pen	August 2017
Xmatkuil (XM)	Merida, Yucatan	20°51′54.9″N, 89°37′24.8″W	Secondary forest	September 2017
Parque Aak (PA)	Xcunya, Yucatan	21°07′48.1″N, 89°37′16.5″W	Secondary forest	November 2018
Hunucma (HU)	Hunucma, Yucatan	21°02′46.4″N, 89°53′19.7″W	Secondary forest	January 2019
Sotuta (ST)	Sotuta, Yucatan	20°39′28.3″N, 89°02′30.1″W	Pasturage	March 2019
Dzemul (DZ)	Dzemul, Yucatan	21°12′48.1″N, 89°19′36.2″W	Secondary forest	March 2019
David Gustavo (DG)	Bacalar, Quintana Roo	18°54′02.8″N, 88°42′31.5″W	Secondary forest	September 2019
Zoh Laguna (ZL)	Calakmul, Campeche	18°35′17.7″N, 89°25′05.7″W	Near a natural pond	September 2019
Dzoyaxche (DO)	Merida, Yucatan	20°47′17.10″N, 89°35′27.66″W	Cave	November 2020
Homún (HO)	Homun, Yucatan	20°44′24.94″N, 89°17′08.24″W	Cave	December 2020
El Remate (ER)	Calkini, Campeche	20°30′25.2″N, 90°23′03.0″W	Near a natural pond	December 2020
Calcehtok (CA)	Opichen, Yucatan	20°33′02.5″N, 89°54′44.4″W	Cave	February 2021
Sitilpech (SI)	Izamal, Yucatan	20°56′23.00″N, 88°57′26.00″W	Near an animal pen	March 2022

The infected hosts were deposited in the Colección Zoológica (CZ), Campus de Ciencias Biológicas y Agropecuarias, Universidad Autónoma de Yucatán. Catalogue numbers are provided in Supplementary Table S1.

### Collection and morphological identification of helminths

All collected organs were dissected from each bat and immersed in distilled water in Petri dishes using a stereo microscope (Olympus SZ2-ILST). Helminths were collected, counted and preserved in 70% ethanol until definitive morphological and molecular identification. For morphological characterizations, nematodes were cleared and temporarily mounted in lactophenol; platyhelminths were stained with carmine acid, dehydrated through an ethanol series, cleared in methyl salicylate and mounted permanently in Canada balsam. Specimens were studied under light microscopy (Leica DM500). Morphological features were used to identify helminths at different taxonomic levels (e.g. order, family and genus), using keys for nematodes (Anderson *et al*., 2009), cestodes (Khalil *et al*., 1994) and trematodes (Jones *et al*., 2005; Bray *et al*., 2008), as well as specialized literature on helminths of bats (e.g. Falcón-Ordaz *et al*., 2006; Caspeta-Mandujano *et al*., 2017). Helminth voucher specimens were deposited in the Colección Nacional de Helmintos (CNHE), Instituto de Biología, Universidad Nacional Autónoma de México. Catalogue numbers are included in Supplementary Table S2.

### Statistical analysis

The prevalence and mean intensity of infection for each helminth taxon were estimated according to Bush *et al*. (1997) and calculated using Quantitative Parasitology 3.0 software (Rózsa *et al*., 2000). The 95% confidence intervals for both parameters were calculated (Rózsa *et al*., 2000; Reiczigel, 2003).

### DNA extraction and sequencing of helminths

Total genomic DNA of some helminths was extracted using the DNeasy Blood & Tissue Kit (Qiagen, Hilden, Germany) following the manufacturer's instructions. The 28S gene of ribosomal DNA was amplified and sequenced using a conventional polymerase chain reaction (PCR) with the forward primer 391 5′-AGCGGAGGAAAAGAAACTAA-3′ (Stock *et al*., 2001) and the reverse primer 536 5′-CAGCTATCCTGAGGGAAAC-3′ (Stock *et al*., 2001), which amplify a fragment of 1400 base pairs. These fragments were amplified using PCR protocols and thermal profiles previously described (Hernández-Mena *et al*., 2017; Panti-May *et al*., 2021).

28S PCR products were sequenced (Laboratorio de Secuenciación Genómica de la Biodiversidad y de la Salud, Instituto de Biología, Universidad Nacional Autónoma de México, Mexico) with the previously used primers (391 and 536) and the following internal primers: 503 5′-CCTTGGTCCGTGTTTCAAGACG-3′ (García-Varela and Nadler, 2005) and 504 5′-CGTCTTGAAACACGGACTAAGG-3′ (Stock *et al*., 2001). The resulting sequences were analysed and edited in Geneious Pro 4.8.4 software (Biomatters Ltd., Auckland, New Zealand) and a consensus was obtained for each sequenced specimen. Sequences generated in this study were submitted to GenBank (National Center for Biotechnology Information). Accession numbers are given in Supplementary Table S3.

### Phylogenetic analysis

To corroborate the identification and genealogical relationships of the parasites, phylogenetic analyses were performed with the new DNA sequences. The alignments of the sequences were generated with ClustalW (http://www.genome.jp/tools/clustalw/) using the approach ‘SLOW/ACCURATE’ and weight matrix ‘CLUSTALW (for DNA)’ (Thompson *et al*., 1994). The nucleotide substitution model was estimated for each dataset with jModelTest v2 (Darriba *et al*., 2012). The phylogenetic analyses were performed with the maximum-likelihood method (ML) in RAxML v. 7.0.4, and executed with 1000 bootstrap repetitions to obtain the best phylogenetic tree of each dataset (Stamatakis, 2006). The trees were visualized and edited in FigTree v. 1.4.4. The molecular variation of 28S datasets was calculated using *P*-distances with software MEGA v6 (Tamura *et al*., 2013).

### Host–parasite network analysis

Host–parasite analysis was used to explore interactions by focusing on the level of host species (pooled host species at all study sites) (Paladsing *et al*., 2020). A host–parasite matrix (presence/absence data, with host species in rows and helminth species in columns) was created using the ‘vegan’ (Oksanen *et al*., 2020) and ‘bipartite’ packages (Dormann *et al*., 2021) in R freeware (R Core Team, 2021) v. 4.0.4. A bipartite network graph was generated with the ‘plotweb’ function showing infections in each host species. Network modularity was also computed using the ‘computeModules’ function and illustrated using the ‘plotModuleWeb’ function (Paladsing *et al*., 2020). The mean number of interactions per species and the mean number of shared organisms were estimated using the function ‘grouplevel’.

## Results

### Helminth fauna of collected bats

A total of 163 bats of 21 species belonging to 6 families (Emballonuridae, Phyllostomidae, Mormoopidae, Noctilionidae, Molossidae and Vespertilionidae) were examined (Table 2). Parasitized bats were recorded in all the studied families except in Vespertilionidae.

**Table 2.** Bats sampled for this study in the Yucatan Peninsula

Family	Species	Capture site	Guild trophic	Number of examined hosts
Emballonuridae	*Peropteryx macrotis*	HO	I	2
	*Saccopteryx bilineata*	ER	I	4
Phyllostomidae	*Desmodus rotundus*	PA, DO, SI	S	6
	*Glossophaga mutica*	HP, PN, XM, HU, DZ	N	9
	*Mimon cozumelae*	DZ, DO	I	5
	*Carollia sowelli*	HP	F	2
	*Sturnira parvidens*	HP, PA, HU, ST	F	8
	*Artibeus jamaicensis*	HB, HP, PN, XM, PA, DZ, ZL	F	48
	*Artibeus litutarus*	HP, PN, ZL	F	3
	*Chiroderma villosum*	HP, PN	F	6
	*Dermanura phaeotis*	HB, DG, ZL	F	8
	*Uroderma bilobatum*	HP	F	1
Mormoopidae	*Mormoops megalophylla*	DG, ZL, CA	I	14
	*Pteronotus fulvus*	HB, CA	I	8
	*Pteronotus mesoamericanus*	HP, DO	I	9
Noctilionidae	*Noctilio leporinus*	ER	P	4
Molossidae	*Eumops nanus*	ER	I	1
	*Molossus nigricans*	HB, HO	I	11
	*Nyctinomops laticaudatus*	HO, CA	I	12
Vespertilionidae	*Eptesicus furinalis*	DG	I	1
	*Rhogeessa aeneus*	PN	I	1
	Total			163

I, insectivore; S, sanguinivore; N, nectarivore; F, frugivore; P, piscivore.

Following morphological characterization and molecular analyses of the 28S gene, 20 helminth taxa were recorded: 7 trematodes Lecithodendriidae gen. sp., *Nudacotyle quartus* (Nudacotylidae), *Limatulum* sp. 1, *Limatulum* sp. 2 (Phaneropsolidae), *Pygidiopsis macrostomum* (Heterophydae), *Urotrema minuta* (Pleurogenidae) and *Brachylecithum* sp. (Dicrocoeliidae); 3 cestodes *Vampirolepis* sp. 1, *Vampirolepis* sp. 2 and *Vampirolepis* sp. 3 (Hymenolepididae) and 10 nematodes Spirurida fam. gen. sp., Strongylida fam. gen. sp., Capillaridae gen. sp., *Pseudocapillaria* sp. 1, *Pseudocapillaria* sp. 2, (Capillaridae), Anoplostrongylinae gen. sp., *Linustrongylus pteronoti*, *Tricholeiperia* cf. *proencai*, *Anoplostrongylus* sp. and *Biacantha desmoda* (Molineidae). Among these, 14 helminth taxa could not be morphologically identified at the genus or species level due to various reasons, such as the inadequate fixation of some specimens, the finding of only a single specimen, and the limited number of bat-associated helminth sequences in GenBank.

### Infection levels

Forty-four (26.9%) bats of 12 species from 5 families were parasitized by helminths. Among these, 31 (70.4%) harboured 1 helminth species, 9 (20.4%) had 2 helminth species and 4 (9.1%) were infected by 3 parasite species.

The prevalence of each helminth species ranged from 7.1 (Anoplostrongylinae gen. sp.) to 100% (Spirurida fam. gen. sp., *Limatulum* sp. 1 and *U*. *minuta*) but in general, it did not exceed 50%. The mean intensity of infection ranged from 1 (e.g. *Pseudocapillaria* sp. 2, Lecithodendriidae gen. sp.) to 56 (e.g. *Limatulum* sp. 2), but in general it was higher for digeneans (Table 3).

**Table 3.** Infection levels of helminths of bats from the Yucatan Peninsula, Mexico

Helminth taxa	Host (*n*)	P (CI)	MI (CI)	SI
Nematoda				
Spirurida fam. gen. sp.	*E. nanus* (1)	100	3 (3)	S
Strongylida fam. gen. sp.	*P. mesoamericanus* (9)	11 (2–48)	1 (1)	I
Anoplostrongylinae gen. sp.	*M. megalophylla* (14)	7.1 (0.2–33)	1 (1)	I
*Linustrongylus pteronoti*	*P. fulvus* (8)	12.5 (0.3–52)	1 (1)	I
*Pseudocapillaria* sp. 1	*M. megalophylla* (14)	50 (23–76)	2.9 (1.9–3.7)	I
	*P. mesoamericanus* (9)	22 (2–60)	1.5 (1–1.5)	I
*Pseudocapillaria* sp. 2	*P. fulvus* (8)	12.5 (0.3–52)	1 (1)	I
Capillaridae gen. sp.	*M. nigricans* (11)	27 (6–60)	1.3 (1–1.7)	S
*Tricholeiperia* cf. *proencai*	*N. leporinus* (4)	50 (6–93)	6.5 (3–6.5)	I
*Anoplostrongylus* sp.	*N. laticaudatus* (12)	50 (21–78)	6.5 (2.3–12.2)	I
*Biacantha desmoda*	*D. rotundus* (6)	50 (11–88)	9 (2–14.3)	I
Cestoda				
*Vampirolepis* sp. 1	*G. mutica* (9)	22 (2–60)	3 (1–3)	I
*Vampirolepis* sp. 2	*M. megalophylla* (14)	21 (4–50)	8 (4–10)	I
*Vampirolepis* sp. 3	*N. laticaudatus* (12)	66 (34–90)	2.6 (1.6–4)	I
Trematoda				
Lecithodendriidae gen. sp.	*P. macrotis* (2)	50 (12–98)	1 (1)	I
*Nudacotyle quartus*	*A. jamaicensis* (48)	8 (2–19)	15 (5.3–24.5)	I, L
	*C. villosum* (6)	33 (4–77)	41 (1–41)	I
*Limatulum* sp. 1	*P. fulvus* (8)	50 (15–84)	8.5 (2.5–19)	I
*Limatulum* sp. 2	*E. nanus* (1)	100	56 (56)	I
*Pygidiopsis macrostomum*	*N. leporinus* (4)	50 (6–93)	45 (34–45)	I
*Urotrema minuta*	*E. nanus* (1)	100	15 (15)	I
	*N. laticaudatus* (12)	25 (5–57)	7 (2–11)	I
*Brachylecithum* sp.	*N. laticaudatus* (12)	16.7 (2–48)	3 (2–3)	L

P, prevalence; MI, mean intensity, CI, 95% confidence interval values; SI, anatomical site of infection; L, liver; S, stomach; I, intestine.

### Phylogenetic analysis

The results of the phylogenetic analyses confirmed that the 28S gene sequences derived from specimens identified morphologically as *T*. cf. *proencai* and *P*. *macrostomum* were identical to the published sequences for these parasites from Campeche (Panti-May *et al*., 2021). On the other hand, the 28S sequences of the *Vampirolepis* sp. 1, *Vampirolepis* sp. 2, *Vampirolepis* sp. 3, *Brachylecithum* sp. and *U*. *minuta* specimens confirmed their identities at the genus or species level while for *Anoplostrongylus* sp., *Pseudocapillaria* sp. 1, *N*. *quartus*, *Limatulum* sp. 1 and *Limatulum* sp. 2, the obtained 28S sequences represent the first available genetic data.

The 28S sequences of *T*. cf. *proencai* and *Anoplostrongylus* and *Pseudocapillaria* sp. 1 were analysed separately in 2 data matrices due to genetic divergences between trichostrongylins and capillariids. The phylogenetic tree of Trichostrongylina grouped *T*. cf. *proencai* with another sequence of the same species from Campeche while *Anoplostrongylus* sp. was grouped as sister species of *T*. cf. *proencai* (bootstrap = 100) and they were nested in the same subclade with other species of the families Heligmonellidae and Trichostrongylidae, although with low support values (bootstrap = 33) (Supplementary Fig. S1). The genetic difference between *T*. cf. *proencai* and *Anoplostrongylus* sp. was 8%. For Capillaridae, the resulting phylogenetic tree showed that our sequence of *Pseudocapillaria* sp. 1 was nested in a subclade formed by *Capillaria plica*, *Aonchotheca paranalis*, *Capillaria* sp. and *Baruscapillaria* sp., with low support values (bootstrap = 40) (Supplementary Fig. S2). The genetic distances between *Pseudocapillaria* sp. 1 and the other capillariids were more than 40%.

**Fig. 2.**
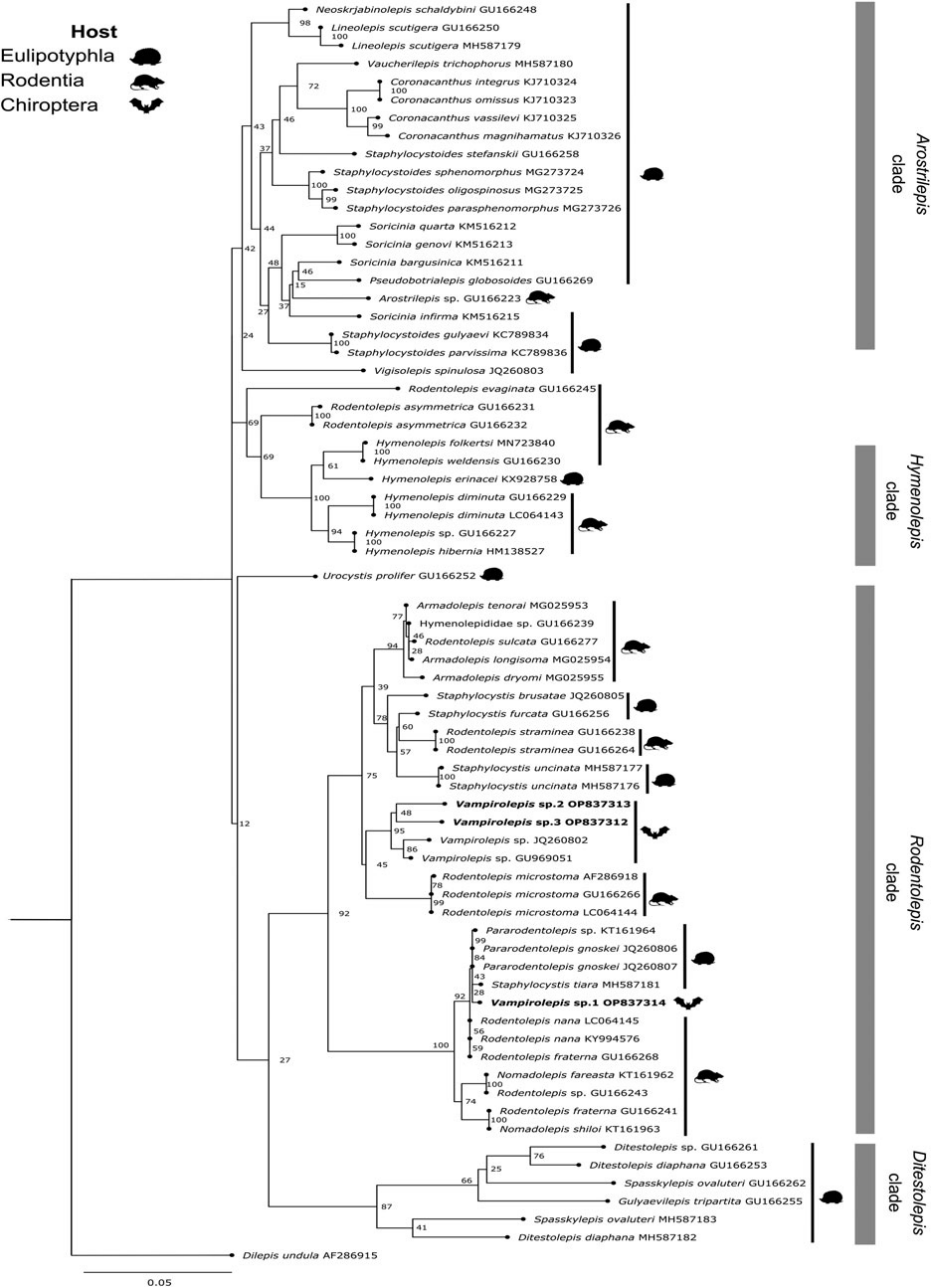
Phylogenetic tree based on the ML analysis constructed on partial large subunit ribosomal gene (28S) of hymenolepidids from different mammalian hosts (likelihood = −9474.057208). Grey bars mark hymenolepidid clades recognized by Haukisalmi *et al*. (2010) and Neov *et al*. (2019). The new sequences of the present study are in bold.

The 28S sequences of *Vampirolepis* from Mexico were aligned with other 65 sequences belong to the family Hymenolepididae. The tree obtained was divided into 4 main clades, similar to those proposed by Haukisalmi *et al*. (2010) and Neov *et al*. (2019). Our specimens of *Vampirolepis* sp. 2 and *Vampirolepis* sp. 3 were grouped as sister species and nested with other specimens of *Vampirolepis* from bats in Finland and China with high support values (bootstrap = 95), while the specimen identified as *Vampirolepis* sp. 1 was nested with sequences of *Pararodentolepis*, *Rodentolepis* and *Staphylocystis* from rodents and eulipotyphlads (bootstrap = 100) (Fig. 2). The genetic distances between our 3 *Vampirolepis* sequences ranged from 2.2 to 4.6%.

**Fig. 3.**
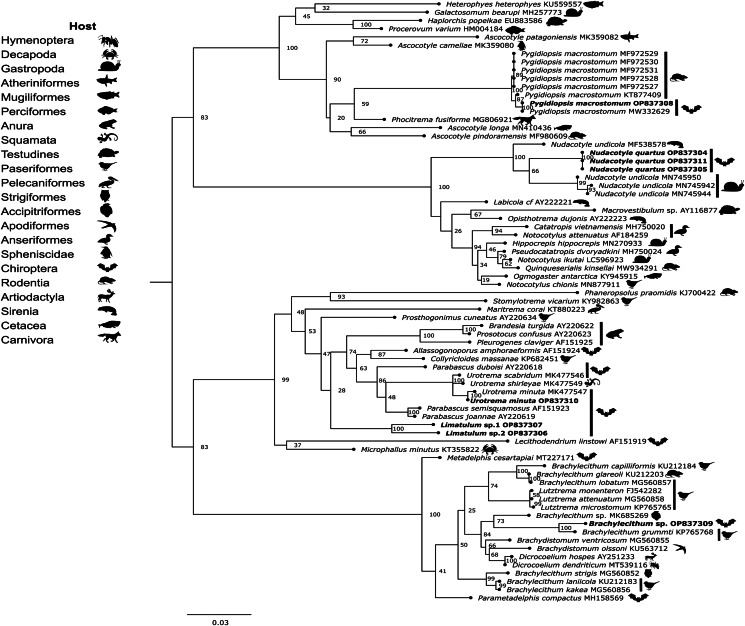
Phylogenetic tree based on the ML analysis constructed on partial large subunit ribosomal gene (28S) of Trematoda species from different hosts (likelihood = −13 934.207182). Some of the sequences included in the analysis were obtained from larvae of the intermediate hosts. The new sequences of the present study are in bold.

The alignment of Trematoda dataset comprised of 73 sequences, including sequences of *P*. *macrostomum*, *N*. *quartus*, *U*. *minuta*, *Limatulum* sp. 1, *Limatulum* sp. 2 and *Brachylecithum* sp. from the Yucatan Peninsula. Our specimens were grouped into 4 main clades (Fig. 3). The first clade grouped our sequence of *P*. *macrostomum* with other sequences of the same species (bootstrap = 100), and nested within the clade formed by other members of Heterophyidae with high support values (bootstrap = 100). The second clade included the families Nudacotylidae, Labicolidae, Opisthotrematidae and Notocotylidae (bootstrap = 100). The sequences of *N*. *quartus* were nested with other nudacotylids (bootstrap = 100) and had genetic distances that ranged from 2 to 4.9% with respect to the larva of *Nudacotyle undicola* from its intermediate host *Biomphalaria pfeifferi* in Kenya. The third clade grouped species from the families Lecithodendriidae, Stomylotrematidae, Prosthogonimidae, Pleurogenidae, Phaneropsolidae and Microphallidae (bootstrap = 100). In this clade, our specimens were grouped into 2 different subclades: the first included *U*. *minuta* and other species of *Urotrema* (bootstrap = 100) while the second subclade grouped *Limatulum* sp. 1 and *Limatulum* sp. 2 (bootstrap = 100). The genetic differences between our specimen of *U*. *minuta* and another sequence of the same species from *Lasiurus seminolus* in the USA, and between *Limatulum* sp. 1 and *Limatulum* sp. 2 were 0.3 and 4.4%, respectively. Finally, the 4th clade comprised members of the family Dicrocoeliidae. The sequence of *Brachylecithum* sp. was grouped as sister species of *Brachylecithum grummti* from *Attila cinnamomeus* in Brazil (bootstrap = 100); the genetic distance between these species was 2.1%.

### Host–parasite network analysis

The interactions between 12 species of bats (hosts) and 20 taxa of helminths (parasites) were explored through group indices, bipartite network and modularity graphs. The mean number of interactions per species was 1.2 for helminths and 2.4 for bats while the average number of shared organisms for helminths was 0.08 and 0.04 for bats.

Three helminth species were found in 2 bat species. *Urotrema minuta* was recorded in *Eumops nanus* and *Nyctinomops laticaudatus*, *N*. *quartus* in *Artibeus jamaicensis* and *Chiroderma villosum*, and *Pseudocapillaria* sp. 1 in *Mormoops megalophylla* and *Pteronotus mesoamericanus*. Six species of bats (*Peropteryx macrotis*, *Glossophaga mutica*, *Desmodus rotundus*, *Pteronotus fulvus*, *Noctilio leporinus* and *Molossus nigricans*) showed segregated species occurrence and were infected by different helminths than other bats such as *A*. *jamaicensis*, *C*. *villosum*, *M*. *megalophylla*, *P*. *mesoamericanus*, *E*. *nanus* and *N*. *laticaudatus*.

**Fig. 4.**
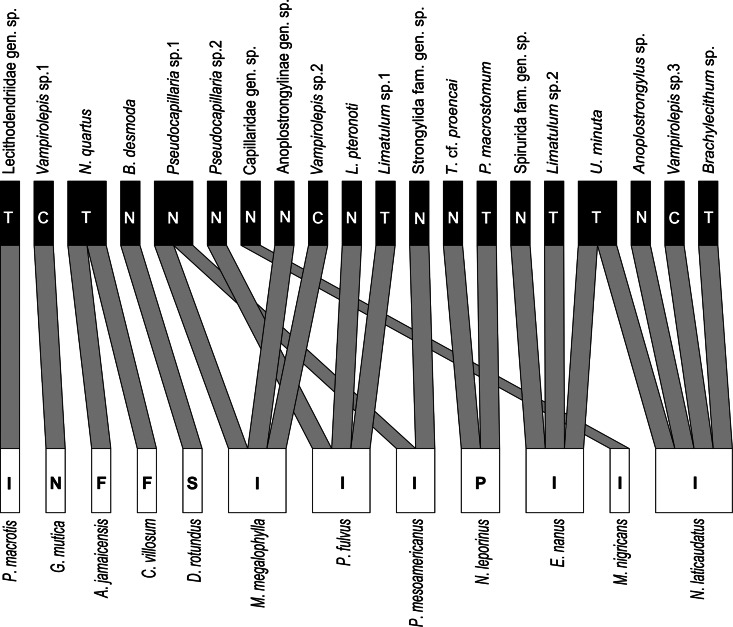
Bipartite network graph illustrating the interactions of helminths of bats from the Yucatan Peninsula, Mexico, based on presence–absence data (helminths: T = trematode, C = cestode, N = nematode; bats: I = insectivore, N = nectarivore, F = frugivore, S = sanguinivore, P = piscivore).

*Nyctinomops laticaudatus* was the only species parasitized by 4 helminth taxa, including cestodes, nematodes and trematodes. In contrast, in 6 bat species only 1 taxon was found (e.g. *P*. *macrotis* infected by Lecitodendridae gen. sp.) (Fig. 4).

**Fig. 5.**
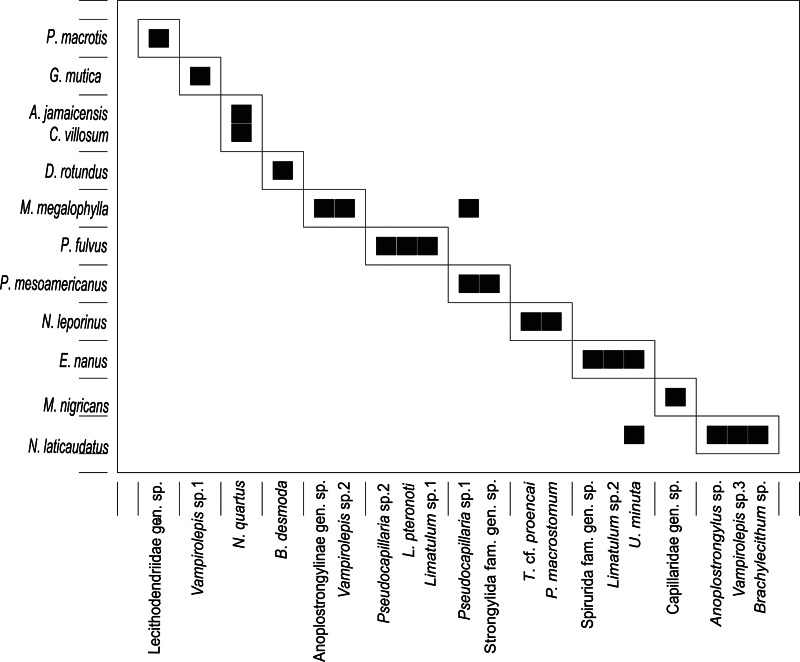
Graph of the bipartite network that illustrates the modules that form in the parasite–host network of helminths of bats from the Yucatan Peninsula, Mexico, based on presence–absence data.

The bipartite network identified 11 modules (network modularity = 0.81) of parasite–host association. In each module, the helminths they contained were associated with 1 host species except in 1 module, which contained *N*. *quartus* in both *A*. *jamaicensis* and *C*. *villosum* (Fig. 5).

## Discussion

### Helminth fauna of collected bats

This large-scale survey is the first to use an integrative taxonomy approach (morphological characters and phylogenetic analysis) to identify the helminth fauna of bats from Mexico. This allowed us to record 20 helminth taxa of bats from the Yucatan Peninsula. Before our work, 14 species had been reported in this region: 11 in Yucatan (Chitwood, 1938; Stunkard, 1938) and 3 in Campeche (Panti-May *et al*., 2021). To the best of our knowledge, the order Spirurida, the genus *Limatulum* and *U*. *minuta* are reported for the first time in *E*. *nanus* in the Americas. This bat occurs from southeastern Gulf of Mexico to Guyana and Peru, but no previous records of helminths have been reported for it (Torres-Morales *et al*., 2014; Santos and Gibson, 2015; Jiménez *et al*., 2017). In addition, *N*. *quartus* is reported for the first time from *C*. *villosum* (Santos and Gibson, 2015; Jiménez *et al*., 2017; Fugassa, 2020), a bat species that occurs from southeastern Mexico to South America (Garbino *et al*., 2020).

In addition, this study reports 6 new helminth–bat associations from Mexico: *Anoplostrongylus* sp., *Brachylecithum* sp. and *U*. *minuta* for *N*. *laticaudatus*, *N*. *quartus* for *A*. *jamaicensis*, *Pseudocapillaria* sp. 1 for *M*. *megalophylla* and *P*. *mesoamericanus*, and *Pseudocapillaria* sp. 2 for *P*. *fulvus*. The genus *Anoplostrongylus* has been previously recorded from *N*. *laticaudatus* in Cuba (Barus and del Valle, 1967) and *Tadarida brasiliensis* and *Eumops perotis* in Brazil (Santos and Gibson, 2015). Mostly species of *Brachylecithum* are parasites of birds, although some infect small mammals (Rodentia, Insectivora and Chiroptera). For example, in bats, *Brachylecithum taiwanense* has been reported in *Hipposideros armiger* from Taiwan (Casanova and Ribas, 2004; Hildebrand *et al*., 2016). Species of *Urotrema* have been described in various mammal and lizard species. In bats, this genus mainly parasitizes insectivorous species from American and African continents (Martínez-Salazar *et al*., 2020). To date, *Urotrema scabridum* is the only species recorded in several species of bats from Mexico (Caspeta-Mandujano *et al*., 2017). The genus *Pseudocapillaria* occurs in the Americas, Europe, Asia and Oceania and includes species that parasitize fishes, reptiles, birds and mammals (Moravec, 1982, 2002). In American bats, *Pseudocapillaria pillosa* has been reported from *Sturnira lilium* and *Lonchophylla robusta* in Brazil and Colombia (Santos and Gibson, 2015).

### Infection levels

The overall helminth infection in bats from the Yucatan Peninsula was 26.9%. When we compared this result with other surveys of multiple species of bats, we observed that most studies reported higher infection values, for example in the USA (37.3–63%) (Pistole, 1988; Hilton and Best, 2000), Mexico (40%) (Salinas-Ramos *et al*., 2017), Peru (56.7%) (Minaya *et al*., 2020), Argentina (61.3%) (Milano, 2016) and Egypt (43.9%) (Saoud and Ramadan, 1976). On the other hand, similar infection levels (20.9–26%) have been reported in bats from Brazil (Nogueira *et al*., 2004; de Albuquerque *et al*., 2016). This variation in helminth infection may be associated with different factors, such as the sample size (Poulin and Morand, 2000), the tropic group of studied bats (Hilton and Best, 2000), the sampling period, season (Salinas-Ramos *et al*., 2017) and the environment surrounding roost sites (Warburton *et al*., 2016).

Although nematodes were the most diverse group of helminths, trematodes had the highest prevalence and mean intensity values. This has been observed in similar studies in Brazil (de Albuquerque *et al*., 2016), the USA (Hilton and Best, 2000), England (Lord *et al*., 2012), Spain (Esteban *et al*., 2001) and Egypt (Saoud and Ramadan, 1976). It is important to point out the occurrence of trematodes in frugivorous bats such as *A*. *jamaicensis* and *C*. *villosum*. Although the feeding ecology of most Neotropical bat species is still poorly known (Nogueira and Peracchi, 2003), the presence of insects in the diet of *A. jamaicensis* has been reported (Ortega and Castro-Arellano, 2001), which could explain the trematode infection by ingestion of infected intermediate hosts. Transmission of trematodes through consumption of water or vegetation contaminated with infective stages is also plausible. Ameel (1944) reported that metacercariae of *Nudacotyle novicia* were immediately infective after encystment on the surface of vegetation and water.

### Phylogenetic analysis

In our study, the phylogenetic position of nematodes was ambiguous with low clade support values due to the limited number of the 28S gene sequences available in GenBank. The generation of subsequent 28S sequences may help to improve the resolution of the phylogenetic relationships of nematodes at supraspecific levels (e.g. genera and families).

The general configuration of our phylogenetic tree of hymenolepidids (Fig. 2) was similar to the previous phylogenetic hypothesis for relationships among members of the family Hymenolepididae from mammals (Haukisalmi *et al*., 2010; Neov *et al*., 2019). We added 3 sequences of *Vampirolepis* from Neotropical bats belonged to the families Phyllostomidae, Mormoopidae and Molossidae. This genus is poorly represented in GenBank; only 2 sequences are available, 1 from Finland and the other 1 from China. Our analysis revealed the same main phylogenetic clades proposed by Haukisalmi *et al*. (2010) and confirmed by Neov *et al*. (2019): *Arostrilepis* clade, *Ditestolepis* clade, *Hymenolepis* clade and *Rodentolepis* clade. The latter contained cestodes of rodents, shrews and bats and diverged in several groups. One of them included our sequences of *Vampirolepis* sp. 2 and *Vampirolepis* sp. 3 and other 2 sequences of *Vampirolepis*. In contrast, *Vampirolepis* sp. 1 was grouped with the genera *Staphylocystis* and *Pararodentolepis* from shrews and rodents. This suggests the non-monophyletic position of the genus *Vampirolepis* due to the distant position of *Vampirolepis* sp. 1 and its relationship with the subclade harbouring the genera *Staphylocystis* and *Pararodentolepis*. The parasite–host patterns across the phylogenetic tree of mammalian hymenolepidids suggest the presence of events of host switching during the process of parasite diversification, including host switching between members of different mammalian orders (Neov *et al*., 2019).

The 28S gene has been extensively sequenced in trematodes (Pérez-Ponce de León and Hernández-Mena, 2019), which allow us to corroborate our morphological identifications at different taxonomic levels. Although the identification of *P*. *macrostomum* and *U*. *minuta* was confirmed by phylogenetic analysis, for other species such as *Brachylecithum* sp. and *N*. *quartus* the corroboration was only up to the genus level. In the case of *Brachylecithum* sp., the ambiguity of some morphological characters (e.g. the length of caeca) made its morphological determination difficult at the genus level; however, the result of the phylogenetic analysis allowed us to position it as a sister species of *B*. *grummti*. Despite the vast 28S sequences of digeneans available in GenBank, no previous sequences of *Limatulum* had been sequenced. The phylogenetic analysis confirms our morphological hypothesis that 2 *Limatulum* species were found in the Yucatan Peninsula.

The increased availability of gene sequences of parasites in the last 2 decades has contributed to the accelerated description of parasite biodiversity. The addition of molecular analyses to traditional morphological descriptions has become part of the accepted best practice to characterize helminth species (Poulin and Presswell, 2016; Poulin *et al*., 2019). In this sense, genetic data can be an information source for the diagnosis and taxonomy of helminths, especially for those groups that present complications in their systematics (e.g. Dicrocoelidae) (Poulin *et al*., 2019; Suleman *et al*., 2020) or when key taxonomic characters cannot be studied due to insufficient or incomplete material (Xu *et al*., 2021). Our results highlight the importance of complementing morphological identification of helminths of Neotropical bats with molecular tools.

### Host–parasite network analysis

In this study, almost all helminths were associated with a specific bat. This result has also been observed by Hilton and Best (2000) in bats from Alabama, USA. The helminth specificity may be associated with the diet of bats, which may predispose a host to be infected with specific helminths and their infective stages (Hilton and Best, 2000; Salinas-Ramos *et al*., 2017). For instance, guppies (Poeciliidae) are the second intermediate hosts of *P*. *macrostomum*, a parasite reported from the piscivorous bat *N*. *leporinus*. The high number of modules indicates marked differences in the helminth fauna even between species of the same trophic guild, which may be associated with factors such as the types of prey (potential intermediate hosts) and the sites of foraging that may vary between species (Hilton and Best, 2000; Clarke-Crespo *et al*., 2017). In addition, this may be related to phylogenetic relationship because more related host species (e.g. from the same family or genus) tend to harbour more similar helminth fauna (Poulin, 2014).

We identified that 3 helminth species occurred in 2 host species, *N*. *quartus* in *A*. *jamaicensis* and *C*. *villosum*, *Pseudocapillaria* sp. 1 in *M*. *megalophylla* and *P*. *mesoamericanus* and *U*. *minuta* in *E*. *nanus* and *N*. *laticaudatus*. This ability of parasites to infect multiple hosts has been linked to coevolutionary relationships, environment, geography and trait matching between host and parasite (Dallas *et al*., 2017). Coevolutionary relationships often lead to host specificity, the degree to which parasites are restricted to particular species of hosts (Poulin, 2010). Closely related hosts may harbour the same parasite because they have similar physiology or immunology or because they provide similar habitats and resources (Presley *et al*., 2015). In our context, the helminth species that were found in more than 1 host, parasitized bats of the same family (i.e. *N*. *quartus* in phyllostomids, *Pseudocapillaria* sp. 1 in mormoopids and *U*. *minuta* in molossids). It has also been noted that parasites with heteroxenous life cycles that include 1 or 2 intermediate hosts have more opportunities to switch hosts (Presley *et al*., 2015). This may arise because free-living infective larvae and infected intermediate hosts containing larval stages may survive for long periods and be exposed to various definitive hosts. Although most investigations of bat-associated helminths have illustrated a dominance of digeneans, most life cycles of this group of parasites remain completely unknown (Blasco-Costa and Poulin, 2017), such as of the Pleurogenidae (Tkach *et al*., 2019).

Insectivorous bats harboured more helminth species in the Yucatan Peninsula. Species such as *N*. *laticaudatus*, *P*. *fulvus*, *M*. *megalophylla* and *E*. *nanus* harboured more helminth taxa compared to bats with other types of feeding such as nectarivores (e.g. *Glossophaga mutica*) and sanguinivores (e.g. *D*. *rotundus*). Similar results have been reported from bats in Brazil and Argentina (de Albuquerque *et al*., 2016; Milano, 2016), which may be associated with the consumption of potential intermediate hosts for cestodes and trematodes (Clarke-Crespo *et al*., 2017; Salinas-Ramos *et al*., 2017). On the other hand, the presence of heteroxenous species in frugivorous (*A*. *jamaicensis* and *C*. *villosum*) and nectarivorous (*G*. *mutica*) bats suggests they may consume insects as a part of their diets or acquire the infection through the incidental ingestion of intermediate hosts.

Our study was limited by the sample size of bats, the sampling period and the use of 1 gene. Nevertheless, our study is the first large-scale survey of helminths of bats that use an integrative taxonomy approach in Mexico. We provided new host records and nucleotide sequences of some helminths (e.g. *N*. *quartus* and *Limatulum* sp. 1) that help us to increase the information available on parasites of bats for future studies. Furthermore, this is the first study that explores the parasite–host interactions in bats in Mexico, which is required to know and understand the transmission patterns of helminths and the relative influence of environmental factors on spatial variation of community composition.

## Conclusion

A high richness (20 taxa) of helminths was found in bats from the Yucatan Peninsula. The prevalence and intensity of infection varied widely. We noted that most helminths parasitize a single host species, and that the insectivorous species had the highest richness of helminths. It would be advisable to conduct further studies examining seasonal and geographical factors as well as intrinsic factors affecting helminth infections. This can help us to increase our knowledge on the helminths parasitizing bats in Mexico and other Neotropical areas.

## Data Availability

All relevant data are within the paper and its supporting information file.

## Supplementary material

For supplementary material accompanying this paper visit https://doi.org/10.1017/S0031182022001627. click here to view supplementary material
