# Supplementary material for: Survey on helminths of bats in the Yucatan Peninsula: infection levels, molecular information and host–parasite networks
Source: Parasitology. 2022 Nov 29;150(2):172–83. doi: 10.1017/S0031182022001627 (PMC10090612; doi:10.1017/S0031182022001627)

**Supplementary material**

Table S1: Accession number of bat symbiotypes deposited at the Colección Zoológica, Yucatan, Mexico.

| Bat species | Catalogue number |
| --- | --- |
| *Artibeus jamaicensis* | 1215, 1221, 1225, 1241 |
| *Chiroderma villosum* | 1212, 1213 |
| *Desmodus rotundus* | 1609, 1610, 1611 |
| *Eumops nanus* | 1612 |
| *Glossophaga mutica* | 1158, 1392 |
| *Molossus nigricans* | 1134, 1135, 1137 |
| *Mormoops megalophylla* | 1471, 1475, 1477, 1479, 1482, 1484, 1563, 1616 |
| *Noctilio leporinus* | 1613, 1614, 1615, |
| *Nyctinomops laticaudatus* | 1472, 1474, 1476, 1478, 1481, 1483, 1485, 1486, 1487, 1488, 1490 |
| *Peropteryx macrotis* | 1489 |
| *Pteronotus fulvus* | 1469, 1470, 1473, 1480 |
| *Pteronotus mesoamericanus* | 1157, 1159 |

Table S2: Accession numbers of vouchers deposited in the Colección Nacional de Helmintos, México.

| Helminth species | Host species | Catalogue number |
| --- | --- | --- |
| *Nudacotyle quartus* | *Chiroderma villosum*, *Artibeus jamaicensis* | 11739, 11740, 11741 |
| *Pygidiopsis macrostomum* | *Noctilio leporinus* | 11742 |
| *Urotrema minuta* | *Eumops nanus*, *Nyctinomops laticaudatus* | 11744, 11745, 11750 |
| *Limatulum* sp. 1 | *Pteronotus fulvus* | 11747, 11748, 11749 |
| *Limatulum* sp. 2 | *Eumops nanus* | 11751, 11752 |
| *Brachylecithum* sp. | *Nyctinomops laticaudatus* | 11746 |
| Lecithodendriidae gen. sp. | *Peropteryx macrotis* | 11743 |
| *Vampirolepis* sp. 1 | *Glossophaga mutica* | 11736 |
| *Vampirolepis* sp. 3 | *Nyctinomops laticaudatus* | 11738 |
| *Vampirolepis* sp. 2 | *Mormoops megalophylla* | 11737 |
| *Tricholeiperia* cf*. proencai* | *Noctilio leporinus* | 11753 |
| *Anoplostrongylus* sp. | *Nyctinomops laticaudatus* | 11754, 11764 |
| *Pseudocapillaria* sp. 1 | *Mormoops megalophylla*, *Pteronotus mesoamericanus* | 11755, 11759, 11765 |
| *Biacantha desmoda* | *Desmodus rotundus* | 11756 |
| Capillaridae gen. sp. | *Molossus nigricans* | 11757 |
| *Pseudocapillaria* sp. 2 | *Pteronotus fulvus* | 11758 |
| Spirurida fam. gen. sp. | *Eumops nanus* | 11760 |
| Anoplostrongylinae gen. sp. | *Mormoops megalophylla* | 11761 |
| *Linustrongylus pteronoti* | *Pteronotus fulvus* | 11762 |
| Strongylida fam. gen. sp. | *Pteronotus mesoamericanus* | 11763 |

Table S3: GenBank accession numbers for the sequences reported in this study.

| Helminths species | Accession numbers |
| --- | --- |
| *Tricholeiperia* cf*. proencai* | OP837303 |
| *Anoplostrongylus* sp. | OP837302 |
| *Pseudocapillaria* sp. 1 | OP837301 |
| *Vampirolepis* sp. 1 | OP837314 |
| *Vampirolepis* sp. 2 | OP837313 |
| *Vampirolepis* sp. 3 | OP837312 |
| *Nudacotyle quartus* | OP837304, OP837305, OP837311 |
| *Pygidiopsis macrostomum* | OP837308 |
| *Urotrema minuta* | OP837310 |
| *Limatulum* sp. 1 | OP837307 |
| *Limatulum* sp. 2 | OP837306 |
| *Brachylecithum* sp. | OP837309 |

Figure S1. Phylogenetic tree based on the Maximum Likelihood analysis constructed on partial large subunit ribosomal gene (28S) of Trichostrongylina from different hosts (likelihood = -4932.055941). The new sequences of the present study are in bold.


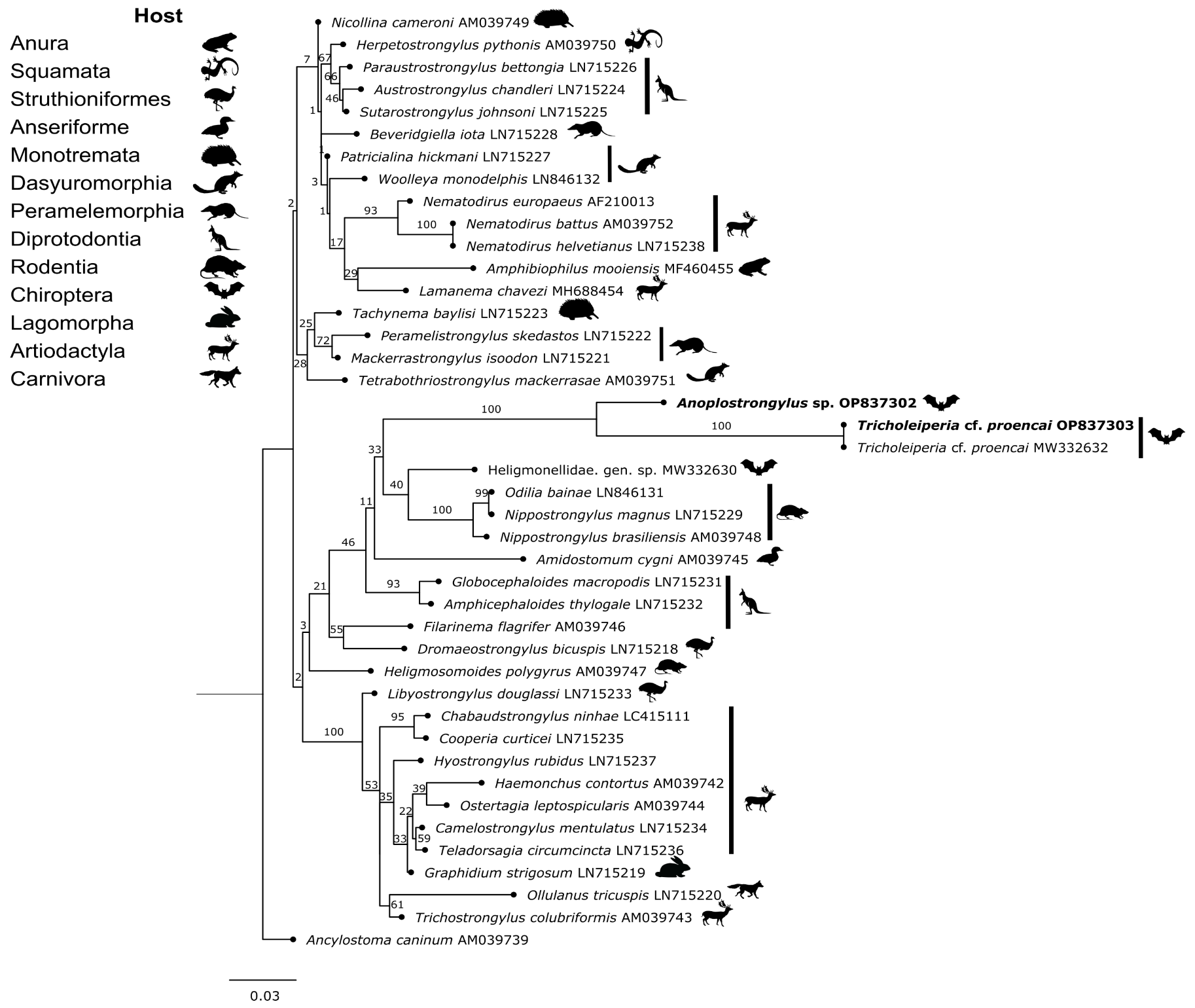


Figure S2. Phylogenetic tree based on the Maximum Likelihood analysis constructed on partial large subunit ribosomal gene (28S) of the family Capillaridae from different hosts (likelihood = -7674.070390). The new sequences of the present study are in bold.


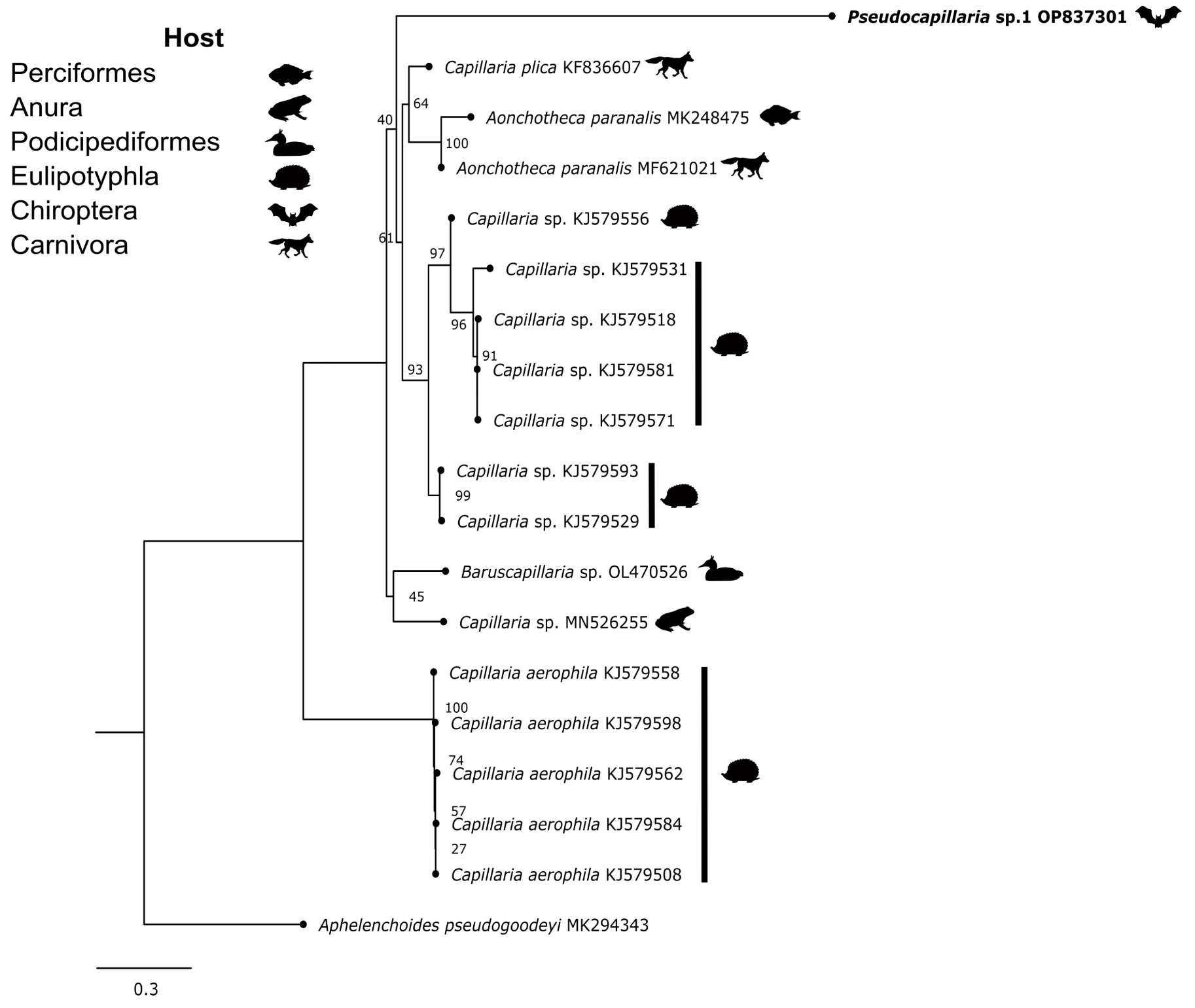

Supplement: Supplementary file 1 [file S0031182022001627sup001.docx]
